# Bookmarking by specific and nonspecific binding of FoxA1 pioneer factor to mitotic chromosomes

**DOI:** 10.1186/1756-8935-6-S1-P94

**Published:** 2013-03-18

**Authors:** Juan Manuel Caravaca, Greg Donahue, Justin S Becker, Kenneth S Zaret

**Affiliations:** 1 Epigenetic Program, Cell and Developmental Biology, Perelman School of Medicine, University of Pennsylvania, Philadelphia, PA 19104-5157, USA

## 

While most transcription factors exit the chromatin during mitosis and the genome becomes silent, a subset of factors remains and “bookmark” genes for rapid re-activation as cells progress through the cell cycle. Yet it is unknown whether such “bookmarking” factors bind to chromatin similarly in mitosis and how different binding capacities among them relate to function. We compared a diverse set of transcription factors involved in liver differentiation and found markedly different extents of mitotic chromosome binding. Among them, only the pioneer factor FoxA1 exhibits virtually complete mitotic chromosome binding. Genomically, about 15% of the FoxA1 interphase target sites are bound in mitosis, including at genes for liver differentiation. Biophysical, genome mapping, and mutagenesis studies of FoxA1 reveals two different modes of binding to mitotic chromatin: Specific binding, at sites that continue to be bound from interphase, and nonspecific binding, whereby due to intrinsic chromatin affinity, FoxA1 remains in the genomic vicinity of its interphase target genes. Both specific and nonspecific binding contribute to timely reactivation of target genes, post mitosis. These studies reveal an unexpected diversity in the mechanisms by which transcription factors help retain cell identity during mitosis.

